# Supplementary material for: VGEA: an RNA viral assembly toolkit
Source: PeerJ. 2021 Sep 6;9:e12129. doi: 10.7717/peerj.12129 (PMC8428259; doi:10.7717/peerj.12129)
Supplement: Supplemental Information 2 [file peerj-09-12129-s002.zip › Supplementary_File_2/Performance_Comparison_Assembly_Pipelines_Table_2/CV45/vgea/quast_results/results_2021_05_13_23_04_04/aligned_stats/cumulative_plot.pdf]

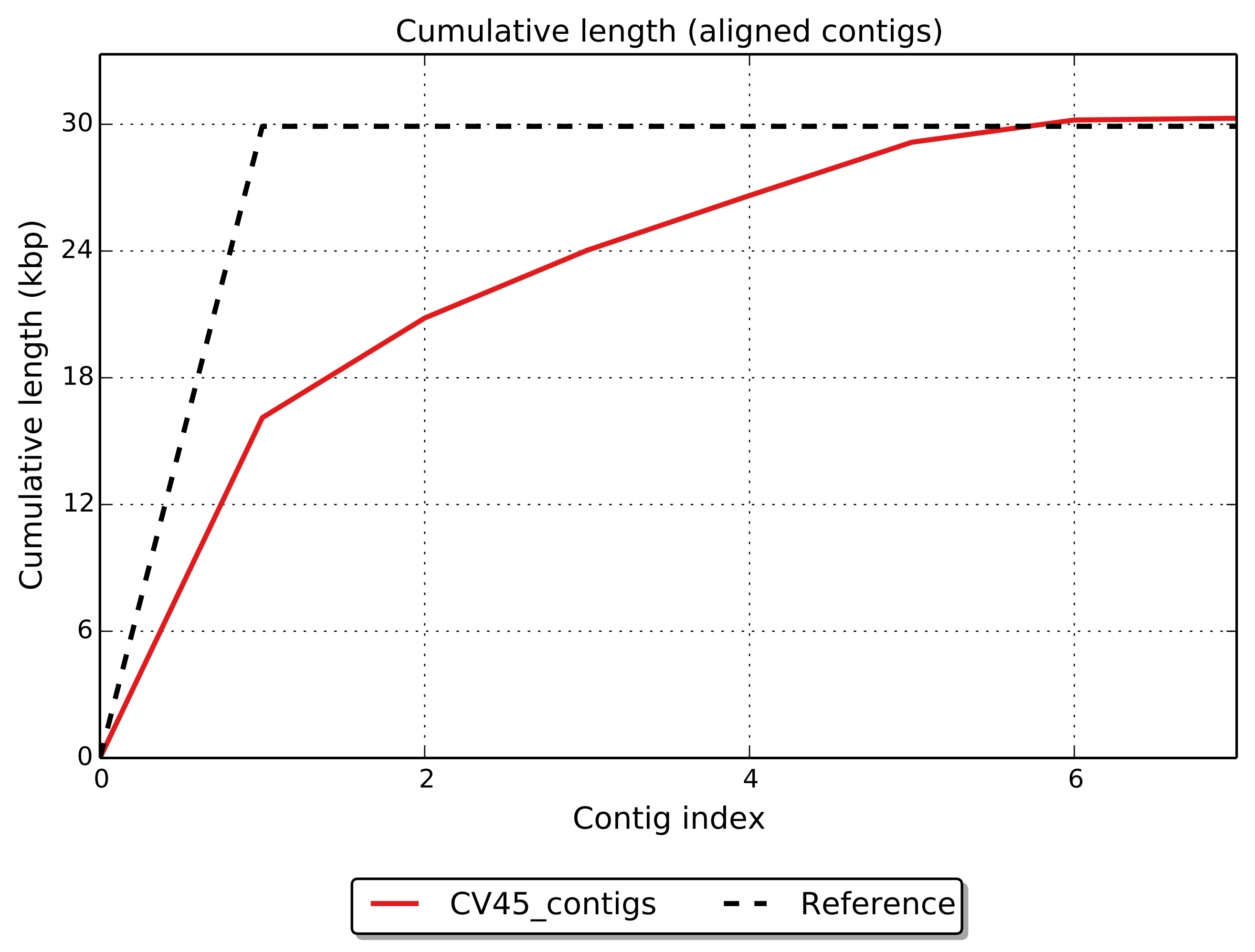
Cumulative length (aligned contigs)
Cumulative length (kbp)
Contig index
0
6
12
18
24
30
0
2
4
6
CV45_contigs
Reference
